# Wastewater bacteria remediating the pharmaceutical metformin: Genomes, plasmids and products

**DOI:** 10.3389/fbioe.2022.1086261

**Published:** 2022-12-16

**Authors:** Betsy M. Martinez-Vaz, Anthony G. Dodge, Rachael M. Lucero, Randy B. Stockbridge, Ashley A. Robinson, Lambros J. Tassoulas, Lawrence P. Wackett

**Affiliations:** ^1^ Department of Biology and Biochemistry Program, Hamline University, St. Paul, MN, United States; ^2^ Department of Biochemistry, Molecular Biology and Biophysics and BioTechnology Institute, University of Minnesota, St. Paul, MN, United States; ^3^ Program in Chemical Biology and Department of Molecular, Cellular and Developmental Biology, University of Michigan, Ann Arbor, MI, United States

**Keywords:** metformin, *Aminobacter*, *Pseudomonas*, genomes, plasmids, guanylurea, gdx, transport

## Abstract

Metformin is used globally to treat type II diabetes, has demonstrated anti-ageing and COVID mitigation effects and is a major anthropogenic pollutant to be bioremediated by wastewater treatment plants (WWTPs). Metformin is not adsorbed well by activated carbon and toxic N-chloro derivatives can form in chlorinated water. Most earlier studies on metformin biodegradation have used wastewater consortia and details of the genomes, relevant genes, metabolic products, and potential for horizontal gene transfer are lacking. Here, two metformin-biodegrading bacteria from a WWTP were isolated and their biodegradation characterized. *Aminobacter* sp. MET metabolized metformin stoichiometrically to guanylurea, an intermediate known to accumulate in some environments including WWTPs. *Pseudomonas* *mendocina* MET completely metabolized metformin and utilized all the nitrogen atoms for growth. *Pseudomonas mendocina* MET also metabolized metformin breakdown products sometimes observed in WWTPs: 1-N-methylbiguanide, biguanide, guanylurea, and guanidine. The genome of each bacterium was obtained. Genes involved in the transport of guanylurea in *Aminobacter* sp. MET were expressed heterologously and shown to serve as an antiporter to expel the toxic guanidinium compound. A novel guanylurea hydrolase enzyme was identified in *Pseudomonas mendocina* MET, purified, and characterized. The *Aminobacter* and *Pseudomonas* each contained one plasmid of 160 kb and 90 kb, respectively. In total, these studies are significant for the bioremediation of a major pollutant in WWTPs today.

## Introduction

Metformin is widely used as a treatment for type II diabetes and is the fourth most prescribed pharmaceutical worldwide (Gong et al., 2012; Briones et al., 2016). This drug is also used to treat other conditions, including polycystic ovarian syndrome, obesity, and most recently, it has been shown to ameliorate symptoms of COVID-19 (Sam and Ehrmann, 2017; Scheen, 2020; Bramante et al., 2021). In addition to lowering blood sugar, metformin has anti-cancer, anti-ageing, and anti-inflammatory properties (Saisho 2015; Novelle et al., 2016; Amin et al., 2019). Recent studies suggest metformin alters the composition of the gut microbiota and that the gut microbiome influences the drug’s therapeutic effects (Wu et al., 2017; Sun et al., 2018; Vich Vila et al., 2020). Because of its widespread use, metformin consumption exceeds 12 billion grams per year globally. The demand for metformin prescriptions is only expected to rise over the next decade due to projected increases in type II diabetes and obesity (Oosterhuis et al., 2013; Bramante et al., 2021; Ibrahim et al., 2021).

In the human body, metformin is only partially metabolized, and it is not completely removed in some water treatment plants (de Jesus Gaffney et al., 2017; Golovko et al., 2021). Metformin and the resulting transformation byproducts are continuously released into aquatic systems and dispersed globally (Scheurer et al., 2012; Tisler and Zwiener, 2018; Elizalde-Velázquez and Gómez-Oliván, 2020). As a result, it has become the most prevalent anthropogenic environmental pollutant in certain surface waters and wastewater treatment plants (WWTPs) worldwide (Scheurer et al., 2012; Briones et al., 2018; Wilkinson et al., 2022). Metformin has a poor affinity for activated carbon, which is the standard method for removing pharmaceuticals. Additionally, metformin chlorination in WWTPs produces N-chloro species that are toxic to human cells (Armbruster et al., 2015; Liao et al., 2021; Zhang et al., 2021; He et al., 2022). While bioremediation has been considered a viable strategy for dealing with metformin contamination, the extent of pathways and enzymes involved in the drug’s microbial metabolism have yet to be elucidated. A knowledge of metformin metabolism is important for understanding its degradation in WWTPs and the drug’s therapeutic mechanism, which has been linked to specific microbiomes in the human gut.

Details on the molecular basis of metformin biodegradation have just recently begun to emerge. Microbial communities from activated sludge have been shown to degrade metformin to guanylurea and an *Aminobacter anthyllidis* strain was shown to convert metformin to guanylurea in a chemostat system (Briones et al., 2016; Poursat et al., 2019; Straub et al., 2019). Two *Pseudomonas* strains were recently isolated on metformin as a nitrogen source and their genome sequences were reported (Hillmann and Niehaus, 2022). We recently reported on the isolation of *Pseudomonas mendocina* GU and determined the genes and enzymes for the mineralization of guanylurea (Tassoulas et al., 2021), a compound that had previously been considered a dead-end metabolite of metformin (Trautwein and Kümmerer, 2011; Trautwein et al., 2014). Guanylurea degradation in strain GU involves a hydrolytic deamination reaction producing guanidine that is catalyzed by GuuH, a novel enzyme of the isochorismate hydrolase-like protein family (Schneider et al., 2020). Guanidine carboxylase, carboxyguanidine deiminase (CgdAB), and allophanate hydrolase (AtzF) complete the degradation of guanidine to carbon dioxide and ammonia *via* a recently recognized biodegradation pathway (Tassoulas et al., 2021). Despite *Pseudomonas mendocina* GU’s ability to grow on various nitrogen-rich compounds (guanidine, agmatine, and urea), this organism was unable to grow on or metabolize metformin and related biguanides.

Here, two very different bacterial strains that degraded metformin were isolated and the genomes sequenced, *Pseudomonas mendocina* GU was subjected to long read sequencing to identify a plasmid, and two deposited plasmids were analyzed. Each isolated bacterium contained a single plasmid, shown here to contain important genes relevant to utilizing metformin. The two new isolates obtained in this study were from different genera and used completely different metabolic strategies in biodegrading metformin.

## Materials and methods

### Enrichment cultures and isolation of pure cultures

Activated sludge samples were collected at the Metropolitan Wastewater Treatment Plant in Saint Paul, Minnesota, United States. Microbial consortia were obtained by enrichment culture with citrate-acetate medium and 1 g of sludge per 50 ml of minimal medium as the inoculum (Aukema et al., 2020; Tassoulas et al., 2021). The minimal medium contained per liter of deionized water: 5.45 g K_2_HPO_4_, 0.2 g MgSO_4_.7 H_2_O, 0.1 g NaCl, 20 mM sodium acetate, and 20 mM sodium citrate (Aukema et al., 2020; Tassoulas et al., 2021). Metformin (Cayman Chemical, Ann Arbor, MI, United States) or 1-N-methylbiguanide (Advanced ChemBlocks, Hayward, CA, United States) (at a concentration of 1 mM) were then added as the sole nitrogen source. When metformin was used as a carbon source, the enrichments contained 3 mM of the drug and 6 mM ammonium chloride as a nitrogen source. Enrichments and isolates were grown at 30°C in a shaking incubator at 200–225 rpm. Cultures were transferred in 10-fold dilutions into fresh medium every 7 days. Individual isolates were obtained by plating 10-fold serial dilutions of the enrichments on selective metformin or 1-N-methylbiguanide plates, transferred to lysogeny-broth (LB) plates, then isolated by streaking on LB and minimal media plus metformin or 1-N-methylbiguanide plates until pure.

### Growth studies

Growth studies were conducted in triplicate. *Pseudomonas mendocina* MET was grown in citrate-acetate media containing 1 mM of each respective nitrogen source: metformin, 1-N-methylbiguanide, biguanide, or guanylurea (Aukema et al., 2020; Tassoulas et al., 2021). Nitrogen-free citrate-acetate media served as a negative control (Aukema et al., 2020; Tassoulas et al., 2021). Citrate-acetate medium containing 6 mM ammonium chloride (NH_4_Cl) was used as a positive control. Cell growth was monitored spectrophotometrically at 600 nm initially at 12–24 h intervals. Cultures with short lag phases were monitored every 4–6 h. *Aminobacter* sp. MET was grown in Ammonium Mineral Salts minimal medium (AMS) composed of 20 mM potassium phosphate buffer (pH 7) plus magnesium sulfate, calcium chloride and trace elements as described (Robinson et al., 2018). Glucose was included as a carbon and energy source at 0.4% and compounds to be used as sole nitrogen sources were provided at 5 mM (metformin, biguanide, methylamine, dimethylamine, guanylurea, or guanidine) or 1 mM (1-N-methylbiguanide). The medium was supplemented with the following vitamin mixture to the indicated working concentrations: biotin, folic acid (1.1 μg/L), 4-aminobenzoic acid, riboflavin (110 μg/L), pantothenic acid, pyridoxine hydrochloride, thiamine hydrochloride, and niacinamide (220 μg/L).

### HPLC identification of degradation products

A Hewlett-Packard (now Agilent Technologies, Santa Clara, CA, United States) 1100 series high performance liquid chromatography (HPLC) system was used to identify substrates and products in growth and degradation assays. Separation of guanylurea from metformin, 1-N-methylbiguanide, and biguanide was achieved by modifying a previously described method (Lin et al., 2020). Sample (10 μl) was injected onto an Agilent Eclipse Plus C18 column (4.6 × 250 mm, 5 μm particle size) and eluted with an isocratic mobile phase of 75:25 (vol:vol) acetonitrile:10 mM potassium phosphate buffer (pH 6.6) at 1 ml/min for 10 min. The latter three compounds could be separated from each other by adjusting the mobile phase to 85:15 (vol:vol) acetonitrile:buffer and extending the elution time to 25 min. Monitoring was done at 200, 220, and 234 nm simultaneously with a diode array detector. Supernatants from growth and degradation assays were passed through 0.2 μm PTFE filters prior to injection onto the column. Assays to detect dimethylamine or monomethyl amine were based on a previously described 9-fluorenylmethoxycarbonyl chloride (FMOC-Cl) derivatization method (Asif Iqbal et al., 2014). Filtered supernatants were combined 3:7 (vol:vol) with 4 mM FMOC-Cl dissolved in acetonitrile, incubated 60 min at room temperature, and then analyzed for the amine-FMOC derivatives (formed by displacement of the chloro group by the amine) by injecting 20 µl of the reaction mixture onto the C18 column and eluting at 1.5 ml/min with an isocratic mobile phase of 7:3 (vol:vol) acetonitrile:deionized water and monitoring at 200 and 262 nm. Peaks in experimental samples were assigned by comparing retention times and UV spectra to those of commercial standards. Quantitation was done using standard curves generated from triplicate analyses of concentration standards prepared for each compound.

### *Aminobacter* sp. MET resting cells assays

Cells were grown on 5 mM metformin as the sole nitrogen source from a starting OD_600nm_ of 0.05 to an OD_600nm_ of 0.5–0.8, harvested by centrifugation, and washed with 1 vol of 1x phosphate buffered saline (PBS). For aerobic assays, cells were resuspended to an OD_600nm_ of 4.0 in PBS containing 0.5 mM metformin and incubated on a shaker at room temperature. Aliquots were removed at time points and analyzed for metformin disappearance and guanylurea appearance by HPLC. For anaerobic assays, 0.5 mM metformin in PBS was first sparged with nitrogen gas and 1.8 ml aliquots were transferred into 2 ml glass vials under a stream of nitrogen. An aliquot of concentrated cell suspension was then added to the vials under a stream of nitrogen to give an OD_600nm_ of 4.0 and the remaining head space was flushed with nitrogen as the vials were sealed with PTFE-lined screw-caps. The reactions were then incubated at room temperature without agitation. Separate reactions were set up for each time point and were analyzed both for metformin and guanylurea or derivatized methylamine and dimethylamine by the HPLC methods described above.

### Genome sequencing and bioinformatics analysis

Total genomic DNA from microbial isolates was sequenced using a Roche GS 454 FLX system and standard LR 70 chemistries. Illumina Nextera XT library preparation and sequencing (on a MiSeq with V3 chemistry and 300 bp paired end reads) services were provided by the Microbial Genome Sequencing Center (SeqCenter, Pittsburgh, PA, United States). Adaptors and low-quality bases were trimmed from raw reads with trimmomatic v 0.36. Long read sequencing was performed using the Oxford Nanopore platform. These sequence reads were used in combination with Illumina reads to improve accuracy at the nucleotide level, assemble plasmids, and for closing genomes. *De novo* assembly was performed using SPAdes v 3.13.0 (Bankevich et al., 2012). Plasmid sequences were identified using plasmidSPAdes and classified using the DoriC 10.0 and OriFinder, databases of replication origins in prokaryotic genomes (Antipov et al., 2016; Luo et al., 2019; Luo and Gao, 2019). The location of the *ori*T in the plasmids, if present, was predicted using *ori*T Finder with Blast E-value cut-off set to 0.01 (Li et al., 2018).

Initial genome and plasmid annotation was performed with Prokka v 1.12 (Seemann, 2014) and manually checked using the genome viewer Artemis (Carver et al., 2012) and Geneious (v11.1.5, URL: http://www.geneious.com) together with blastp. Blastn and tblastx were used for plasmid comparison, using both NCBI tools and within BLAST Ring Image Generator, BRIG v0.95, (URL: http://brig.sourceforge.net/). (Alikhan et al., 2011).

### Gdx protein purification and reconstitution

A synthetic geneblock (Integrated DNA Technologies, Coralville, IA) encoding Gdx-*Aminobacter* was cloned into a pET21b plasmid with a N-terminal hexahistidine tag followed by a Thrombin cleavage recognition site. *Escherichia coli* C43 (DE3) culture was grown to an OD_600nm_ of 0.8 then induced with 0.2 mM isopropyl β -d-1-thiogalactopyranoside (IPTG) for 3 h at 37°C. Cell pellets were re-suspended in 50 mM TRIS-HCl pH 8.5, 100 mm NaCl and disrupted by sonication. Lysate was extracted with 3% (wt/vol) decyl- β -D-maltoside (DM, Anatrace, Maumee, OH) and the soluble fraction was loaded onto a cobalt affinity column (TALON resin, Takara Bio, San Jose, CA) equilibrated with purification buffer (20 mM TRIS-HCl pH 8.5, 500 mm NaCl, 5 mm DM). The column was washed with purification buffer, purification buffer containing 10 mm imidazole, and protein was eluted with purification buffer containing 400 mM imidazole. After buffer exchange to remove imidazole using a PD10 desalting column (GE Healthcare, Chicago, IL, United States), the histidine tag was cleaved using thrombin (MilliporeSigma, Burlington, MA, United States), 1 µl per mg of protein, overnight at room temperature) before a final size exclusion purification step (Superdex200, Cytiva, Marlborough, MA) in 10 mM HEPES pH 8.0, 500 mM NaCl, and 4 mM DM. Freshly purified protein was reconstituted in proteoliposomes as described previously (Kermani et al., 2018; Kermani et al., 2020). Briefly, *E. coli* polar lipids (EPL, Avanti Polar Lipids, Alabaster, AL) were solubilized at a concentration of 20 mg/ml in 100 mM KCl, 100 mM KPO_4_ pH 7.5, 35 mM 3-[(3-cholamidopropyl)- dimethylammonio]-1-propanesulfonate (CHAPS, Anatrace). Protein was added to a final concentration of 40 µg protein/mg lipid (1:25 protein:lipid mass ratio) and detergent was removed by dialysis. Proteoliposomes were stored in aliquots at −80°C until use.

### SSM electrophysiology

SSM electrophysiology experiments were performed as previously described (Bazzone et al., 2017; Kermani et al., 2018)using a SURFE^2^R N1 instrument (Nanion Technologies, Munich, Germany). The SSM sensors were prepared using 1,2-diphytanoyl-sn-glycero-3-phosphocholine (DPhPC) in a non-activating buffer containing 100mM KCl, 100 mM KPO_4_ pH 7.5. Proteoliposomes diluted 25-fold in buffer were sonicated and fused to the sensor lipid layers by centrifugation at 2500 *g* for 30 min. After both the lipidation and proteoliposome fusion steps, the sensor capacitance and conductance were determined using SURFE^2^R software protocols. Only sensors with a capacitance between 15 and 35 nF were used for experiments. For transport measurements, sensors were perfused with test substrate (2 mM) in 100 mM KCl, 100 mM KPO_4_ pH 7.5 buffer, and capacitive currents were recorded. To compare transport currents measured using different sensors, currents were normalized to the peak current generated by the first perfusion of the positive reference substrate, Gdm^+^, measured on that sensor. Data was collected from three independent sensor preparations.

### Guanylurea hydrolase protein expression and purification

The gene for the putative guanylurea hydrolase from *Pseudomonas mendocina* MET (CP098606-CP098607) was codon-optimized for expression in *Escherichia coli* B, synthesized by Integrated DNA Technologies (Coralville, IA), and inserted into pET28b^+^ using an NEBuilder HiFi DNA assembly kit (New England Biolabs, Ipswitch, MA, United States) to include an N-terminal six-histidine tag. The plasmid was purified from *E. coli* DH5α cells with a QIAprep spin miniprep kit (QIAGEN, Hilden, Germany) and the insert was verified by Sanger sequencing (ACTG, Inc., Wheeling, IL, United States). The guanylurea expression strain was created by transforming *E. coli* BL21 (DE3) cells with the plasmid. To express the protein, LB plus 50 μg/ml kanamycin sulfate was inoculated with an overnight culture (1% vol/vol) of the expression strain. The culture was incubated on a shaker at 37°C until reaching an OD_600nm_ of 0.6 and then cooled to 15°C.

Protein expression was induced by adding IPTG to 0.5 mM and incubating at 15°C for 20 h. The induced cells were harvested by centrifugation and stored at −80°C until used. Frozen cells were suspended in 10 ml of lysis buffer (20 mM sodium phosphate, 0.5 M NaCl, pH 7.4), then lysed using a French pressure cell (3 cycles at 140 MPa) and centrifuged at 19,000 × *g* for 90 min. The cleared lysate was loaded into a GE Healthcare (Cytiva, Marlborough, MA) AKTA fast liquid protein chromatography (FPLC) system and injected onto a HisTrap HP 5-ml column (Cytiva) that had been charged with Ni^2+^. Unbound proteins were eluted with lysis buffer containing 20 mM imidazole, weakly bound proteins were eluted with buffer plus 100 mM imidazole, and remaining bound proteins were eluted with a linear gradient from 100 mM to 250 mM imidazole in lysis buffer and a final wash at 500 mM imidazole. Purity of fractions collected during the linear gradient was assessed by SDS-PAGE and then fractions of equivalent purity were pooled and imidazole was removed by five cycles of concentrating the protein to 0.5–1.0 ml with Prometheus (Genesee Scientific, San Diego, CA, United States) centrifugal filters (10,000 molecular weight cutoff) and then diluting to 15 ml with buffer. Protein concentration was measured *via* the Bradford method with the Bio-Rad (Hercules, CA, United States) Protein Assay Dye Reagent Concentrate and a standard curve prepared from a commercial bovine serum albumin (BSA) standard (Thermo Scientific, Rockford, IL, United States) and purity was assessed by SDS-PAGE.

### Guanylurea hydrolase activity assays

Activity of the guanylurea hydrolase was determined by detection of ammonia released from substrates using the colorimetric Berthelot reaction (Weatherburn, 1967). Reactions were performed in triplicate at room temperature in 0.5 ml of 125 mM sodium phosphate buffer (pH 8.0) with 1 mM of substrate added. The amount of enzyme added was 0.5, 400, or 800 μg with guanylurea, biuret, or 2-imino-4-thiobiuret, respectively. Total incubation times were 20 min (4 time points), 6 h (3 time points) or 24 h (2 time points), respectively.

## Results and Discussion

### Metformin-degrading bacteria isolations

A bacterium from a metformin consortium was isolated as a pure culture that grew on metformin as both a carbon source and a nitrogen source. Another bacterium was isolated that grew with metformin only as a nitrogen source, additional carbon sources such as citrate or acetate were required. The cultures were indicated to be pure as evidenced by a single colony type after repeated streaking on agar plates (Supplementary Figure S1) and by finding only one 16S rRNA gene by whole genome sequencing. Each isolate was subjected to metabolic studies, genome sequencing, bioinformatic analyses, protein isolations and protein characterizations. Those studies revealed that the two new metformin-degrading bacteria utilized different strategies for metabolizing metformin.

### Identification and metabolic characterization of *Aminobacter* sp. MET

The 16S rRNA sequencing identified the bacterium using metformin as a carbon source to be an *Aminobacter* species. The DSMZ Type Strain Genome Server identified the isolate to be most related to different species of the genus *Aminobacter* and so we have designated this strain as *Aminobacter* sp. MET (Meier-Kolthoff et al., 2022).

**FIGURE 1:**
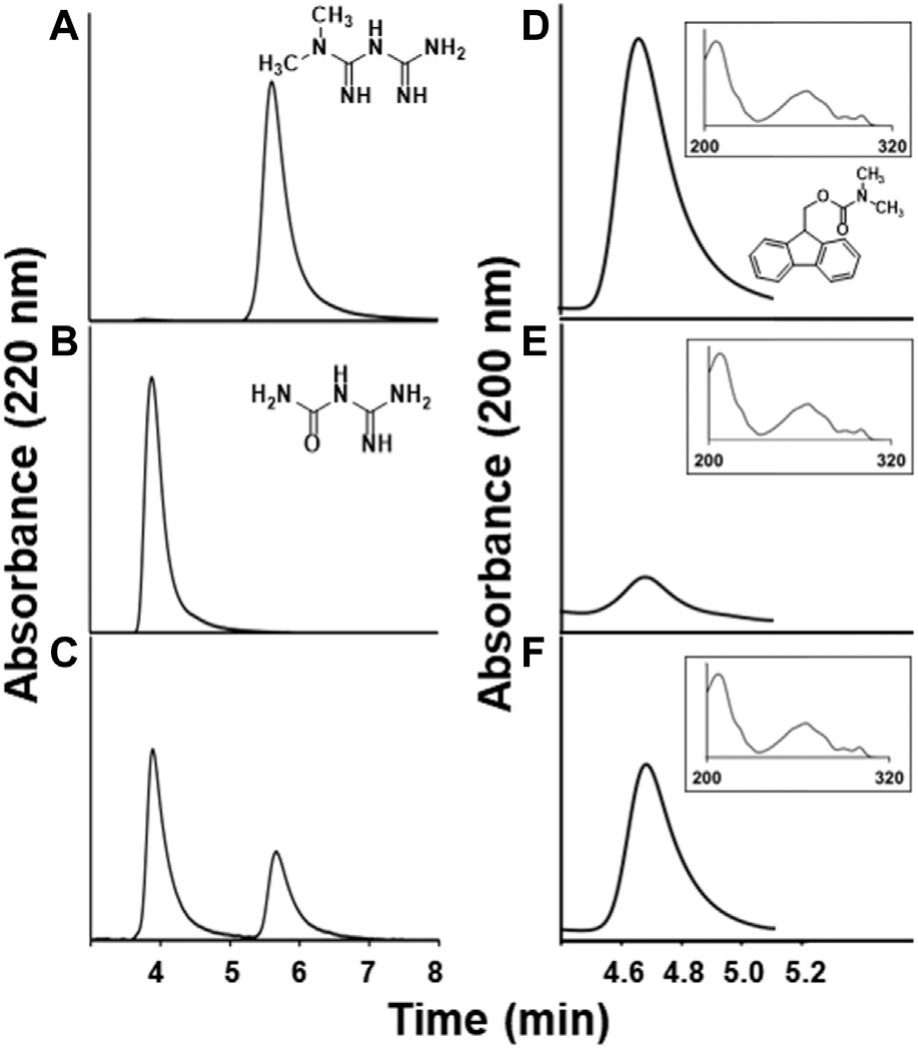
Metformin degradation products in *Aminobacter* sp. MET resting cell assays. HPLC chromatograms showing **(A)** metformin and **(B)** guanylurea standards, **(C)** disappearance of metformin and appearance of guanylurea after 40 min aerobic incubation, **(D)** 0.5 mM dimethylamine standard derivatized with 9-fluorenylmethoxycarbonyl chloride (FMOC-Cl) (derivative structure shown), and putative dimethylamine-FMOC derivatives in anaerobic incubations of metformin with cells after **(E)** 20 min or **(F)** 120 min. The *y*-axis represents the absorbance intensity at a wavelength of 220 nm; the x-axis shows the retention time of the individual compounds eluting from the HPLC column. Inset panels in D-F are UV spectra of the peaks shown (composite of spectra taken at five time points across the peaks) and the inset *x*-axes represent wavelength in nm.

*Aminobacter* sp. MET grew to a final OD_600nm_ of 2.3 with metformin as the sole nitrogen source and to OD_600nm_ of 2.8 with methylamine or dimethylamine as sole nitrogen after 3 days. There was no substantial growth (OD_600nm_ ≤ 0.2) with 1-N-methylbiguanide, biguanide, guanylurea or guanidine supplied as the nitrogen source, even after incubation for 7 days. *Aminobacter* sp. MET also grew on metformin as a sole carbon source. Metformin was shown by HPLC to be consumed and guanylurea increased concomitantly (Figures 1A–B–C). In nitrogen deficient minimal media to which metformin was provided, *Aminobacter* sp. MET supported the growth of *Pseudomonas mendocina* GU. Previously, *Pseudomonas mendocina* GU was demonstrated to use guanylurea as a nitrogen source, but it was unable to metabolize metformin.

The transformation of metformin to guanylurea could occur *via* one or two initial demethylation reactions followed by methylamine or ammonia displacement, respectively. Alternatively, dimethylamine might be displaced directly and its detection in media would argue against initial metformin demethylation. Under aerobic conditions, dimethylamine could not be identified (<0.02 mM). Dimethylamine is reported to be oxidized by a monooxygenase in other *Aminobacter* strains (Artuso et al., 2021) and this could explain the failure to detect it here. In an effort to trap dimethylamine if it were formed, resting cell suspensions were incubated with metformin under anaerobic conditions and then combined with a derivatizing reagent (FMOC-Cl). Near stoichiometric levels of dimethylamine were detected (Figures 1D–E–F). The derivative obtained from cell media matched the retention time of a derivatized standard and showed the same UV-visible spectrum (Asif Iqbal et al., 2014). The methylamine-FMOC derivative, which had a different retention time from the dimethylamine derivative, was not detected (<0.02 mM).

The genome of strain *Aminobacter* sp. MET was analyzed for genes involved in dimethylamine and methylamine metabolism, since dimethylamine monooxygenase produces methylamine. Genome annotation identified two gene clusters, associated with the oxidation of dimethylamine and methylamine, respectively (Table 1). The first cluster is for the multicomponent dimethylamine monooxygenase enzyme system and the second is for enzymes of the N-methyl glutamate (NMG) pathway. Both produce a methylene carbon at the formaldehyde oxidation level and the serine cycle genes were identified in the genome (Table 1).

**TABLE 1:** Enzymes relevant for the metabolism of biguanide, guanidine, nitrogen, and carbon in five different bacterial strains investigated in this study.

Organism	Replicon	Known proteins encoded	Known protein function	Relevant proteins identified	Possible function
*Aminobacter* sp. MET	pMET-1			SugE	Guanylurea export
				SugE	Guanylurea export
				PuuA	Glutamylputrescine synthesis
				HupA	Nickel uptake
				GbuA	Guanidinobutyrate metabolism
				HutG	-N-C=NH group hydrolysis
				HypA	Hydrogenase maturation
				HypB	Hydrogenase maturation
				CodB	Cytosine permease
	Chromosome	DmmABCD	Dimethylamine Monooxygenase		
		GmaS, MgsABC, MgdABCD, FolD, PurU, FdhGBACD	^a^Methylamine glutamate pathway and CH2 = THF oxidation		
		HprA, Gck, Eno, Ppc, Mdh, MtkAB, Mcl, Sga, GlyA	^b^Serine cycle for HCHO assimilation		
*Pseudomonas mendocina* MET	pMET-2	^d^GuuH	^c^Guanylyurea - > guanidine	PuuA	Glutamylputrescine synthesis
				HupA	Nickel uptake
				GbuA	Guanidinobutyrate metabolism
				HutG	-N-C=NH group hydrolysis
				HypA	Hydrogenase maturation
				HypB	Hydrogenase maturation
				CodB	Cytosine permease
	Chromosome	GC, CgdAB, AtzF	^d^Guanidine -> 3 NH_3_	SugE	Guanidinium transport
*Pseudomonas mendocina* GU	pGU				
	Chromosome	GC, CgdAB, AtzF	Guanidine -> 3 NH_3_	SugE	Guanidinium transport
*Pseudomonas* sp. KHPS1	pKHPS1	^d^GuuH	Degradation of guanylyurea		
	Chromosome	GC, CgdAB, AtzF	Guanidine -> 3 NH_3_	SugE	Guanidinium transport
*Pseudomonas hydrolytica* KHPS2	pKHPS2	^d^GuuH	Degradation of guanylyurea		
	Chromosome	GC, CgdAB, AtzF	Guanidine -> 3 NH_3_	SugE	Guanidinium transport

^a^ Methylamine glutamate pathway and CH2 = THF, oxidation enzymes: GmaS glutamate-methylamine ligase; MgsABC, N-methylglutamate synthase subunits; MgdABCD, N-methylglutamate dehydrogenase subunits, FolD, 5,10 methylene tetrahydrofolate dehydrogease/methenyl tetrahydrofolate cyclohydrolase, PurU formyl tetrahydrofolate deformylase; FdhGBACD, NAD-dependent formate dehydrogenase subunits.

^b^ Serine cycle enzymes: GlyA, serine hydroxymethyltransferase; Sga, serine glyoxylate aminotransferase; hpr, Hydroxypyruvate reductase, gck, Glycerate 2-kinase; Eno, enolase; Ppc phosphoenolpyruvate carboxylase; Mdh, malate dehydrogenase; MtkAB, malate-CoA ligase; Mcl, malyl-CoA, lyase.

^c^ Guanylurea and guanidine degradation enzymes: GuuH, guanylurea hydrolase, GC, guanidine carboxylase, CgdAB, carboxyguanidine deiminase, AtzF, allophanate hydrolase.

^d^ Guanylurea hydrolase identified in this study.

**FIGURE 2:**
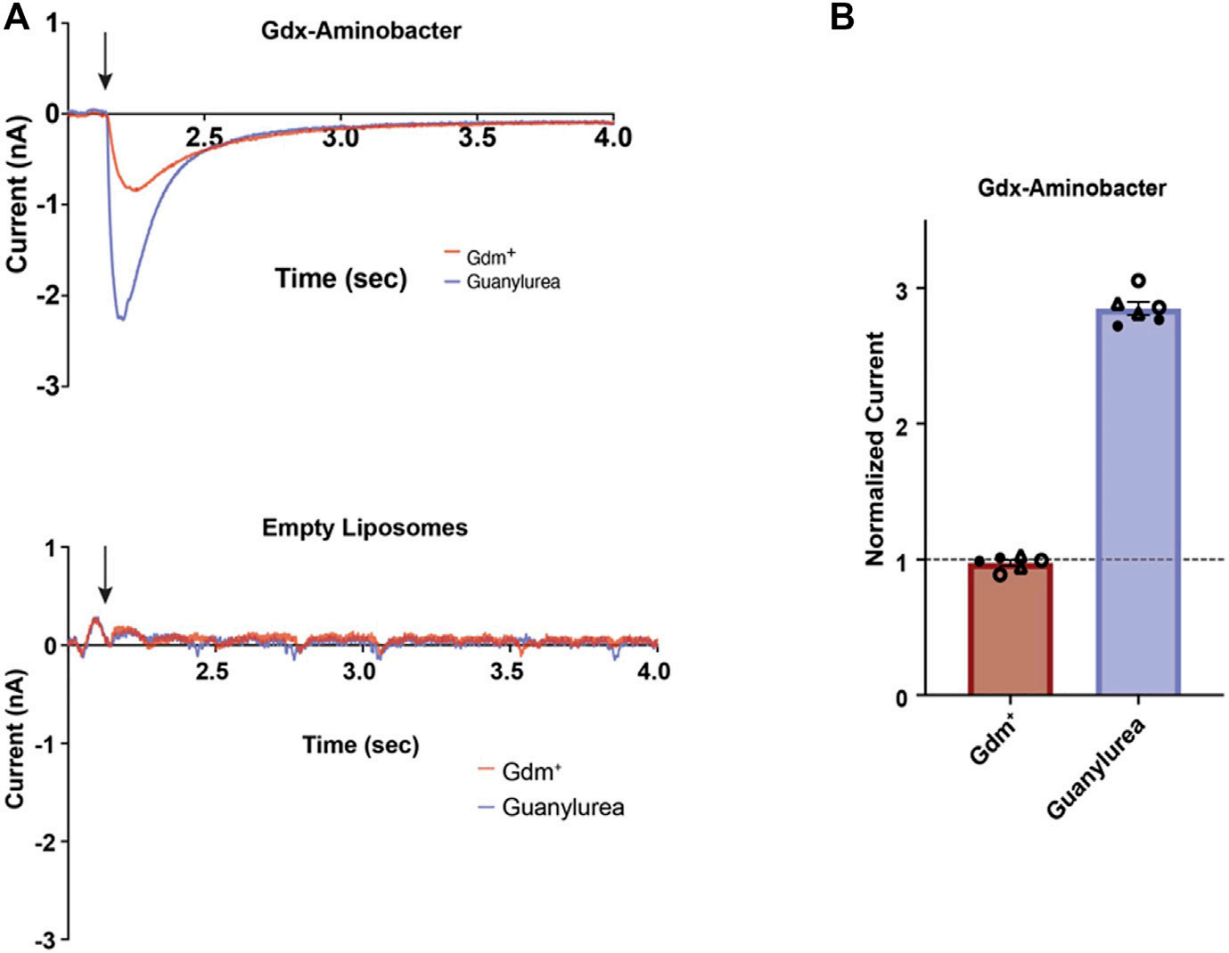
Substrate transport by *Aminobacter* Gdx. **(A)** Representative SSM electrophysiology traces for Gdx-*Aminobacter* proteoliposomes (top) and protein-free liposomes (bottom) upon perfusion (indicated by arrow) with 2 mM Gdm+ (red) or guanylurea (purple). **(B)** Relative amplitude of Gdx-*Aminobacter* peak currents evoked by Gdm+ and guanylurea. Duplicate substrate perfusions were performed for each of three independent sensor preps (independent sensor preps represented by open circles, closed circles, and triangles). Currents were normalized against the first Gdm + trace collected on the same sensor. Error bars represent the mean and SEM of replicate measurements.

Guanylurea was found in the growth medium and may be toxic inside the cell and so its transport was investigated. As a charged molecule, it would need a dedicated mechanism to translocate from inside the cell into the medium. Genome sequencing had identified two contiguous and identical genes encoding SugE family proteins known as Gdx transporters, that export quaternary amine and guanidinium compounds, although none, to our knowledge, had previously been shown to transport guanylurea (Chung and Saier, 2002; Burata et al., 2022). To assess whether the plasmid-borne *Aminobacter* Gdx transporter exports guanylurea, we heterologously expressed, purified, and reconstituted this protein in proteoliposomes (Supplementary Figure S2). We then used solid-supported membrane (SSM) electrophysiology to monitor the movement of charged substrate across the liposome membranes (Figure 2). Like other Gdx transporters (Kermani et al., 2018; Kermani et al., 2020), Gdx-*Aminobacter* exhibited negative capacitive currents upon perfusion with 2 mM guanidinium ion (Gdm^+^), consistent with the electrogenic antiport of >1 H^+^ per transported Gdm^+^. Perfusion with 2 mM guanylurea elicited an even more robust response. Peak currents, which correspond to the initial rate of transport, are ∼3-fold larger for guanylurea than for Gdm^+^.

For sensors prepared without protein, no currents were observed upon perfusion of either substrate. These experiments establish proton-coupled guanylurea antiport by *Aminobacter* Gdx.

In the physiological context, the proton gradient maintained by bacteria would favor export of guanylurea. These transport data are consistent with the HPLC determination of guanylurea produced by *Aminobacter* sp. MET (Figures 1A–B–C) that also was shown to support growth of *Pseudomonas mendocina* GU that grows on guanylurea but not metformin. Our observations here are also consistent with multiple earlier reports that metformin is metabolized to guanylurea as a “dead-end” product in some wastewater treatment systems (Trautwein and Kümmerer, 2011; Trautwein et al., 2014).

### Second enrichment yielded a bacterium metabolizing metformin completely to ammonia

Sludge from the same WWTP was sampled 6 months after the initial enrichment that yielded *Aminobacter* sp. MET. The second enrichment yielded a bacterium able to use metformin as a nitrogen source but not as a carbon source. 16S rRNA analysis identified this bacterium as a *Pseudomonas mendocina*. A significant number of other genes in the isolate were 100% identical to *Pseudomonas mendocina* GU, a bacterium previously shown to degrade guanylurea to carbon dioxide and ammonia (Alanjary et al., 2019; Tassoulas etr al., 2021). Based on these observations, the new strain was designated *Pseudomonas mendocina* MET.

Metformin disappearance from the medium was observed by HPLC but neither guanylurea nor other organic products were discernible. The bacterium also grew on 1-N-methylbiguanide, biguanide, guanylurea, or guanidine as the sole nitrogen source to an OD_600nm_ of 1.0 or greater. To examine whether the bacteria were utilizing all the nitrogen available in these substrates and converting it to ammonia to support growth, we carried out ammonia stoichiometry experiments. Complete metabolism of metformin or biguanide would release 5 equivalents of ammonia. Parallel cultures of *Pseudomonas mendocina* MET were grown with limiting concentrations of metformin, biguanide or ammonium chloride and cell densities were determined when growth ceased. Plotting the data as nitrogen equivalents (Supplementary Figure S3) showed that all five nitrogen atoms from both metformin and biguanide supported growth.

Gene mining identified guanidine carboxylase, carboxyguanidine deiminase (CgdAB), and allophanate hydrolases that were 100% identical at the amino acid level to the corresponding enzymes in *Pseudomonas mendocina* GU, described previously (Tassoulas et al., 2021), and the surrounding genes were also the same (Table 1). However, *Pseudomonas mendocina* MET did not have a gene region that was identical to the one that encodes guanylurea hydrolase in *Pseudomonas mendocina* GU. This observation suggested that guanylurea hydrolase activity was encoded by a different gene.

### Identification of a new guanylurea hydrolase

**FIGURE 3:**
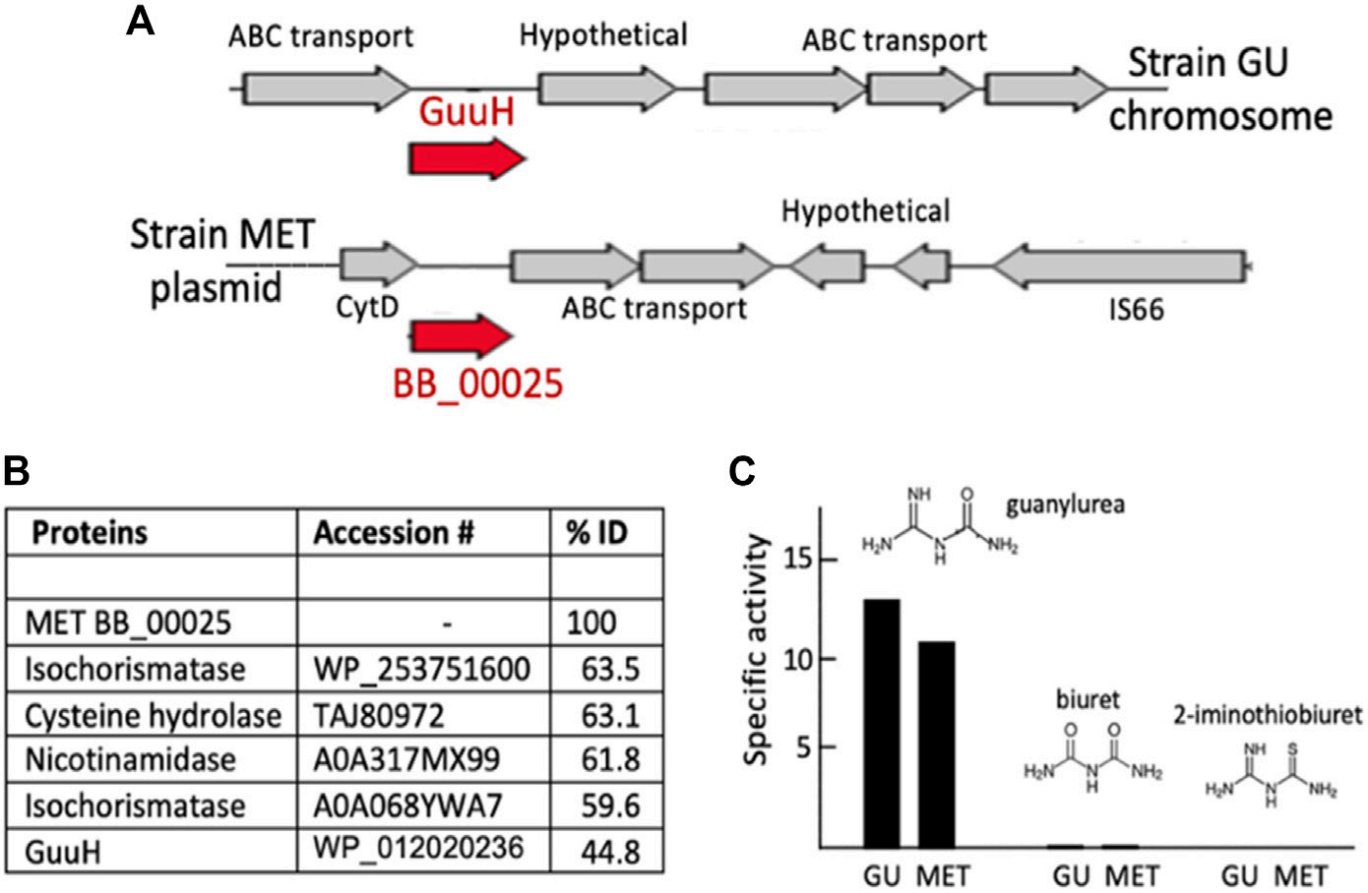
Identification, purification, and characterization of a new guanylurea hydrolase in *Pseudomonas mendocina* MET encoded by the plasmid gene, BB_00025. **(A)** The chromosomal gene region encoding the guanylurea hydrolase (GuuH) of *Pseudomonas mendocina* GU (top) and the plasmid gene region encompassing BB_00025. **(B)** Percent amino acid identitiy comparisons of the amino acid sequences of isochorismatase hydrolase-like (IHL) proteins compared to the translated sequence of the MET strain BB_00025 gene. **(C)** Activity of the BB_00025 gene product (labeled MET) assayed with three substrates and compared to the guanylurea hydrolase (GuuH) from strain GU.

Guanylurea hydrolase is known to be a member of the isochorismatase hydrolase-like (IHL) protein family (PF000857.20). The *Pseudomonas mendocina* MET plasmid (pMET-2) encoded a likely IHL protein (BB_00025) but the gene region was completely different than the previously described GuuH gene region and flanked by insertion elements, suggesting a recent acquisition (Figure 3A). An adjacent gene, annotated as a cytidine deaminase, encodes a metallo-hydrolase that could plausibly react with guanylurea. The IHL protein was selected first to express a synthetic gene and assay the protein. Guanylurea hydrolase proteins share as much as 65% sequence identity with other IHL family enzymes such as nicotinamidase, isochorismatase, biuret hydrolase, triuret hydrolase, and proteins broadly annotated as cysteine hydrolases (Tassoulas et al., 2021). Here, BB_00025 shared >60% sequence identity with enzymes annotated as isochorismatase, cysteine hydrolase and nicotinamidase, but only 44.8% identity to GuuH, the experimentally demonstrated guanylurea hydrolase (Figure 3B). The protein expressed readily in *E. coli* and was purified to homogeneity (Supplementary Figure S4). The protein was assayed with three substrates that structurally resemble guanylurea and are reactive with different members of the IHL superfamily (Figure 3C). The purified BB_00025 protein had a specific activity with guanylurea of 11 μmol/min per mg protein and an apparent *k*_*cat*_ of 4.4 s^−1^, similar to that shown with the reported guanylurea hydrolase, GuuH (Tassoulas et al., 2021). The protein showed very low activity with biuret (0.4 nmol/min per mg, Supplementary Table S1), a property it also shared with GuuH.

The newly identified guanylurea hydrolase in *Pseudomonas mendocina* MET was encoded by a plasmid gene. The three strains compared here, and two plasmids from recently deposited genomes all derived from the same city WWTP. Given the possibility of horizontal transfer spreading metformin-degrading ability, the plasmid sequences were analyzed and compared in greater depth.

### Plasmids in metformin/guanylurea degrading bacteria

**FIGURE 4:**
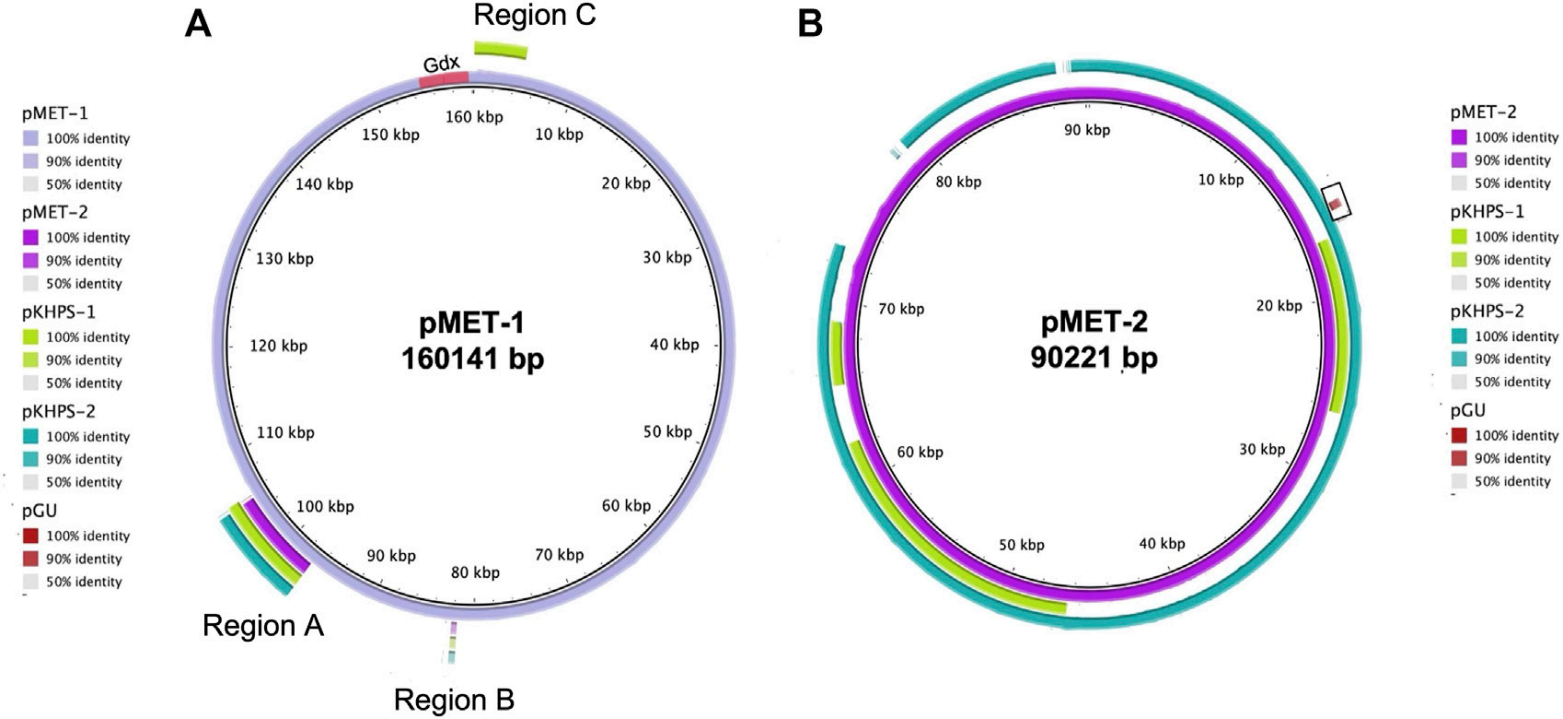
Comparison of plasmids from metformin-degrading isolates using blastn. *Aminobacter* sp. MET (pMET-1) and *Pseudomonas mendocina* MET (pMET-2) were compared with two plasmids from metformin-degraders (pKHPS-1 and pKHPS-2) and a guanylurea degrading bacterium (pGU). Blastn comparisons were visualized using BRIG and the % identity threshold indicated in the panels next to the plasmid’s images (Alikhan et al., 2011). Regions of sequence similarity are represented by outer rings colored on a sliding scale indicating a defined percentage identity (upper and lower identity thresholds of 90% and 50%, respectively). In panel **(A)**, the segments labeled regions A, B, and C represent plasmid DNA fragments containing genes shared by all metformin-degrading bacteria studied. Gdx denotes the guanylurea transporters found in *Aminobacter* sp. MET. The box with the brown segment in panel **(B)** represents a plasmid DNA fragment shared by guanylurea and metformin-degrading *Pseudomonas*.

Overall, one plasmid each was identified in all three strains examined here and a comparison with these and two recently deposited plasmids derived from metformin degraders is shown in Figure 4. The four *Pseudomonas* plasmids ranged in size from 79.8–90.2 kb; the *Aminobacter* plasmid (pMET-1) was much larger, 160.1 kb.

The *Aminobacter* plasmid pMET-1 was predicted to encode 175 open reading frames, approximately 45% of which are hypothetical genes (Supplementary Table S2). Consistent with the results of growth and metabolic studies, we could not identify genes for enzymes involved in guanylurea or guanidine metabolism on plasmid pMET-1, nor on the chromosome of *Aminobacter* sp. MET. Plasmid analysis had identified two identical genes annotated as putative Gdx (guanidinium compound) transporters, shown near the origin of replication at the top of Figure 4A, that were expressed and shown here to transport guanylurea (Figure 2).

The plasmid pMET-1 origin of replication shares 100% nucleotide sequence identity with pR3A (NC_010919), an *Aeromonas hydrophila* plasmid belonging to incompatibility group U (IncU) (Kulinska et al., 2008). Both contain genes for a complete VirB4/VirD4 type IV secretion system (T4SS) with over 95% amino acid sequence identity to T4SSs known to mediate horizontal gene transfer in *Agrobacterium* and *Mesorhizobium* species (Kuldau et al., 1990; Zupan and Zambryski, 1995; Cascales and Christie, 2003; Nelson and Sadowsky, 2015). While pMET-1 lacks F-type conjugative transfer genes, it appears to be a mobilizable plasmid *via* the VirD4-like T4SS genes, a coupling protein and a TraA conjugative transfer relaxase, which are likely involved in nicking at an *ori*T site and unwinding DNA before transfer.

The *Pseudomonas* plasmids were significantly smaller, ranging from 79.8–90.2 kb. The largest plasmid, pMET-2, contains 109 open reading frames, approximately 37% of which encode hypothetical proteins (Supplementary Table S3). The nucleotide sequences of the *Pseudomonas* plasmids were compared using blastn and the similarities are represented as concentric circles using the BLAST Ring Image Generator (BRIG) (Figure 4B) (Alikhan et al., 2011). The pMET-2 plasmid from *Pseudomonas mendocina* MET served as the reference sequence. Regions of sequence identity for the other plasmids are represented on a sliding scale indicating defined percentage identity thresholds (100%, 90%, and 50%, respectively). Plasmid pKHPS-2 is most highly related to pMET-2 with 91% of the genes sharing >90% nucleotide sequence identity. A major difference is pMET-2, being larger, has a unique region of 6,318 bp that encodes hypothetical proteins and a mercury resistance operon. Plasmid pKHPS-1, the smallest at 79,836 bp, shares only 31% of its nucleotide sequence being >90% identical to pMET-2. The plasmid from the guanylurea-degrading strain *Pseudomonas mendocina* GU was substantially different from the plasmids found in metformin-degrading bacteria with only one small region of 436 bp encoding a hypothetical protein of unknown function, highlighted in brown on Figure 4B, in common with pMET-2.

In an effort to learn more about the plasmids present in the metformin-degrading *Pseudomonas* strains, *in silico* plasmid typing was conducted using the pMLST database (Carattoli et al., 2014). All the plasmids from *Pseudomonas* metformin-degraders detailed here have replication-associated genes related to the IncA/C incompatibility group. IncA/C plasmids are large broad host-range, low-copy number extrachromosomal elements with varying conjugation capabilities. Many IncA/C plasmids use type four secretion systems (T4SS) for conjugation and mobilization (Hegyi et al., 2017). Consistent with this observation, all the *Pseudomonas* plasmids described here, encoded a VirB4/VirD4 type IV secretion system (T4SS) with an origin of transfer, a relaxase, and a transfer coupling protein, suggesting they might be self-mobilizable.

Comparative genomic analyses of the four metformin-degrading *Aminobacter* and *Pseudomonas* bacteria identified plasmid regions (Regions A and B) of >90% nucleotide sequence similarity amongst pMET-1, p-MET-2, pKHPS1 and pKHPS2 (Figure 4A). These regions encode ten proteins annotated as urea carboxylase- and cytosine-related transporters and enzymes involved in amino, guanidinium- and formimino-group metabolism. The proteins in region A included a cytosine permease, a formimidoylglutamase (HutG), a guanidinobutyrase (GbuA), two nickel metallochaperones (HypA, HypB), two putative transcriptional regulators, and a hypothetical protein. Region B encoded two urea-carboxylase related ABC transporters that were also conserved amongst all metformin degrading plasmids. An additional region (Region C) consisting of four genes was only common to plasmids pMET-1 and pKHPS1 and was related to a TniABC transposition system.

### Comparisons amongst strains

**FIGURE 5:**
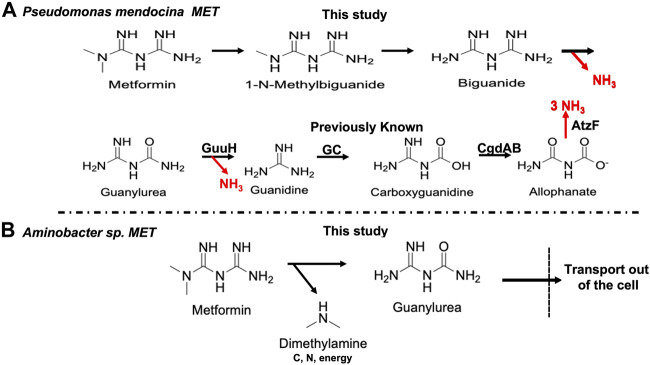
Proposed biodegradation pathways for metformin by **(A)** *Pseudomonas mendocina* MET and **(B)** *Aminobacter* sp. MET. The known enzymes in the pathway are GuuH (guanylurea hydrolase), GC (guanidine carboxylase), CgdAB (carboxyguanidine deiminase) and AtzF (allophanate hydrolase).

These data indicated that *Pseudomonas mendocina* MET metabolizes guanylurea analogously to *Pseudomonas mendocina* GU but, unlike strain GU, grows on metformin, 1-N-methylbiguanide and biguanide, suggesting a plausible pathway for metformin biodegradation (Figure 5A). That proposed pathway provides all five nitrogen atoms to support growth from metformin and biguanide. *Pseudomonas mendocina* MET is not a methylotroph and lacks pathways to assimilate carbon at the formaldehyde level, consistent with our failure to observe growth as a carbon source.

*Aminobacter* sp. MET utilizes a completely different metabolic strategy for metformin degradation (Figure 5B). HPLC, electrophysiology and genomic data support the formation of dimethylamine and guanylurea, with the latter transported outside the cell by Gdx proteins encoded by the plasmid (Figure 4A) and the former metabolized by a known methylated amine pathway *via* chromosomal genes (Table 1). *Pseudomonas mendocina* GU, that does not degrade metformin, had previously been characterized for gene content related to guanylurea metabolism, but a plasmid was not identified in that study. Here, we deduced the plasmid sequence which established that the guanylurea genes are all located chromosomally. This contrasts with strain MET in which the guanylurea hydrolase gene was identified to be on the plasmid and the genes encoding guanidine carboxylase, carboxyguanidine deiminase and allophanate hydrolase are chromosomally localized.

The genes in common to all the metformin degrading bacteria are annotated as: cytosine permease, formimidoylglutamase (HutG), a guanidinobutyrase (GbuA) and two nickel metallochaperones. Currently, research is ongoing to express genes, singly and in combination, in heterologous host organisms to determine their physiological roles.

## Data Availability

The data presented in the study are deposited in the NCBI GenBank Nucleotide repository (https://www.ncbi.nlm.nih.gov/genbank/), accession numbers: JAMSHL000000000, CP098606 and CP098607.

## Author’s note

While our paper was in process, we became aware of a new paper describing a bacterium able to grow on metformin (Chaignaud, P., Gruffaz, C., Borreca, A., Fouteau, S., Kuhn, L., Masbou, J., Rouy, Z., Hammann, P., Imfeld, G., Roche, D. and Vuilleumier, S., 2022. A methylotrophic bacterium growing with the antidiabetic drug metformin as its sole carbon, nitrogen, and energy source. Microorganisms, 10(11), p.2302.). This paper and the present one, illustrate emerging genomic expansions for the metabolism of the pharmaceutical metformin.
