# Supplementary material for: Wastewater bacteria remediating the pharmaceutical metformin: Genomes, plasmids and products
Source: Front Bioeng Biotechnol. 2022 Dec 16;10:1086261. doi: 10.3389/fbioe.2022.1086261 (PMC9800807; doi:10.3389/fbioe.2022.1086261)
Supplement: Supplementary file 2 [file DataSheet1.pdf]

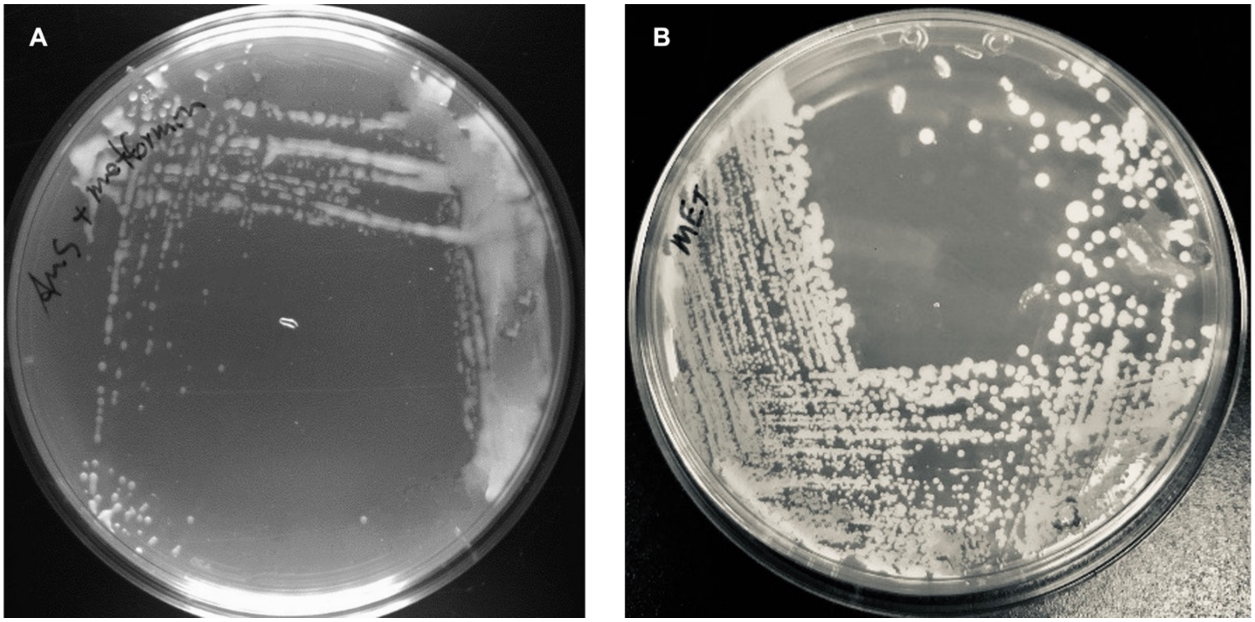

Figure S1: Pure cultures of *Aminobacter* sp. MET (Panel A) and *Pseudomonas mendocina* MET (Panel B) growing on minimal medium with metformin as a sole nitrogen source. Identification of these isolates was done using the sequence of the 16S rRNA genes, and by phylogenetic analyses using the whole genome sequence of each strain.

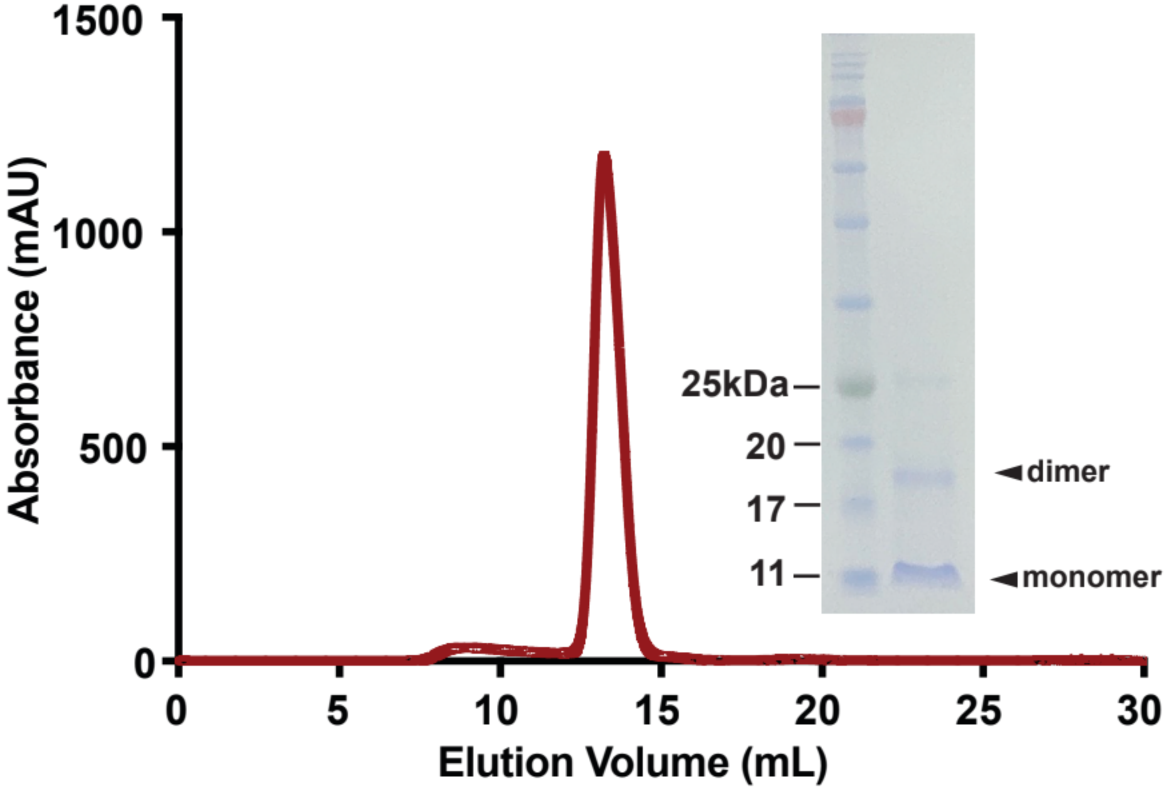

Figure S2: Purification of *Aminobacter* Gdx. Size exclusion chromatogram (SEC) from Gdx-*Aminobacter* purification. Inset: Coomassie-stained SDS-PAGE gel of the main (13 mL) SEC peak. The molecular weight of Gdx-*Aminobacter* is 11.2 kDa. The dimer band shown in the gel is common for membrane proteins that often resist complete denaturation by SDS.

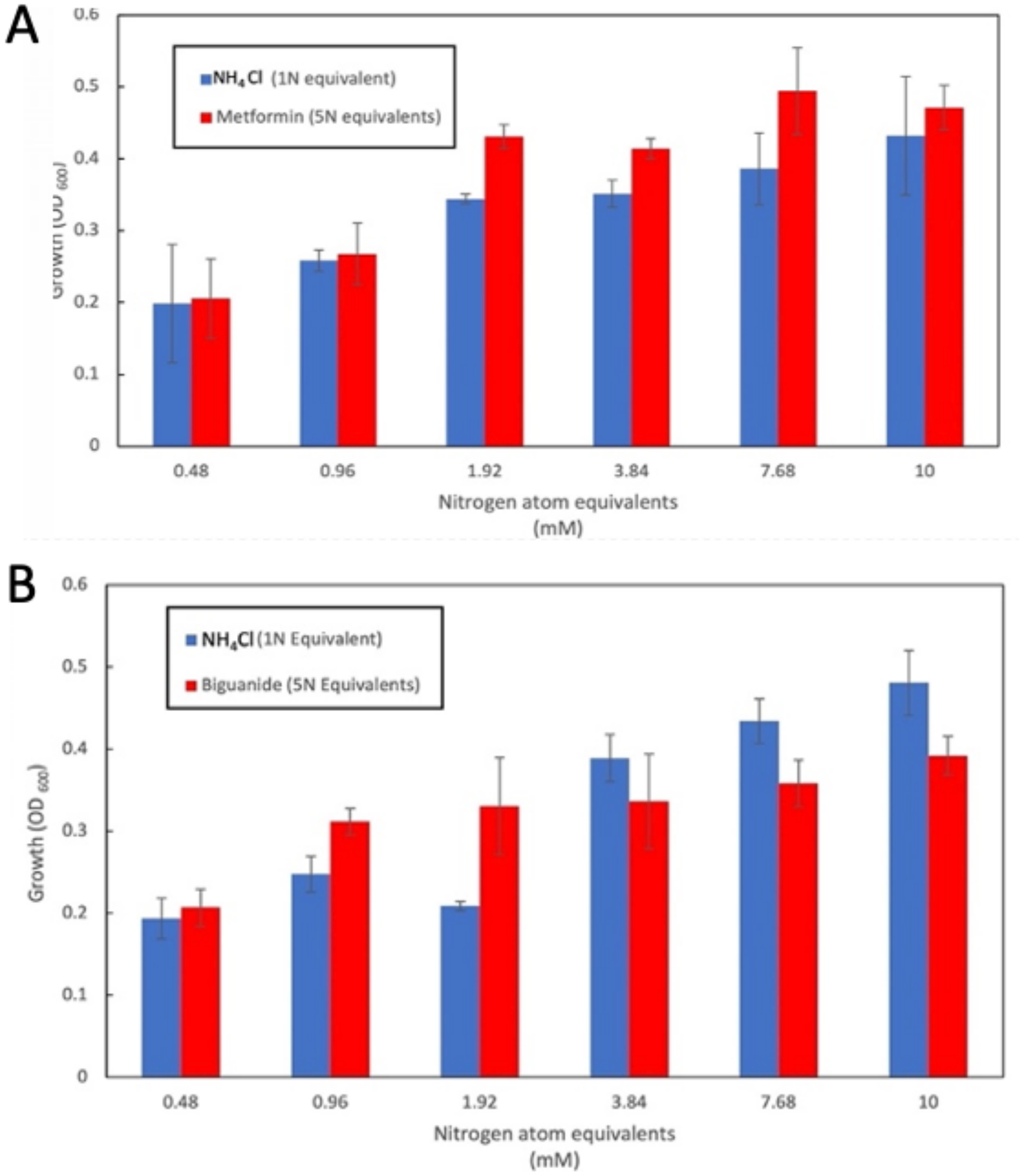

Figure S3: Determination of nitrogen obtained from metformin and biguanide. *Pseudomonas mendocina* MET was grown on minimal medium containing growth-limiting concentrations of ammonium chloride, metformin, and biguanide. The x- axis shows nitrogen equivalents. There are five nitrogen atoms in one metformin or biguanide molecule.

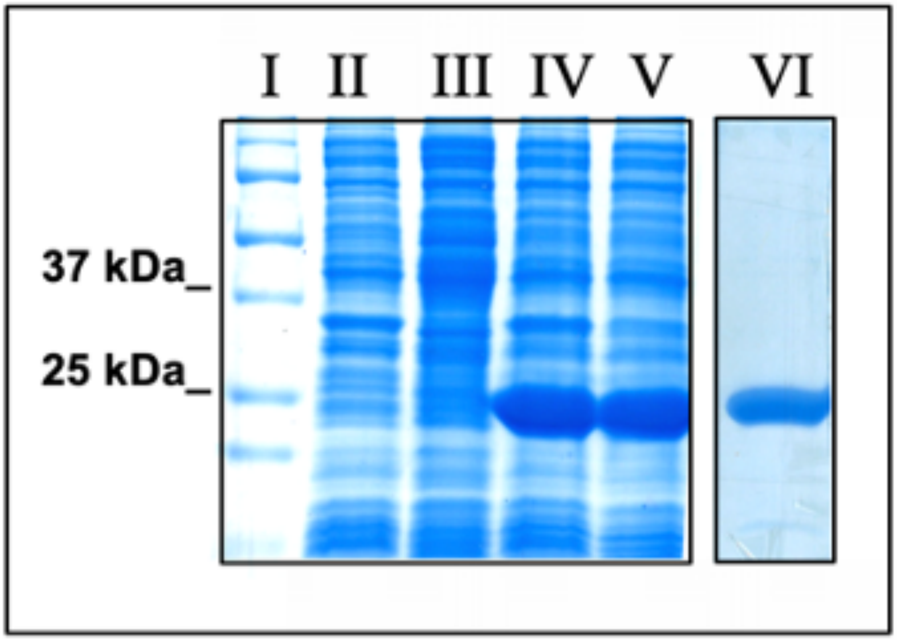

Figure S4: SDS-PAGE analysis of the *Pseudomonas mendocina* MET IHL enzyme overexpressed in *E. coli* BL21(DE3). Lane I is MW standards, II and IV are total protein fractions from the uninduced or induced IHL enzyme expression strain cell lysates, respectively. Lanes III and V are soluble protein fractions from the uninduced or induced IHL enzyme expression strain cell lysates, respectively. Lane VI shows 20 µg of purified IHL enzyme as described in Materials and Methods. Lane VI is a cropped photo of a second gel that was separate from the gel containing lanes I-V.

Table S1. Activity Table for the purified BB_00025 protein and GuuH with 3 substrates

Substrate	Specific activity (μmol min^{-1} mg^{-1})	GuuH (μg)	Time points (min)
Guanylurea	11 ± 1	0.5	5, 10, 15, 20
Biuret	$4 \times 10^{-4} \pm 3 \times 10^{-5}$	200	120, 240, 360
2-Imino-4-thiobiuret	$4 \times 10^{-5} \pm 2 \times 10^{-5}$	400	330, 1440
